# Comparative Study of Cytokeratin Immunostaining of Parotid Gland Parenchyma in Normal, Diabetic, and Excretory Duct Ligation of Mongrel Dogs

**DOI:** 10.1055/s-0042-1744372

**Published:** 2022-06-21

**Authors:** Sherif Sayed Hassan, Mashael Saeed Alqahtani

**Affiliations:** 1 Oral Biology Division, Department of Basic and Clinical Oral Sciences, Faculty of Dentistry, Umm Al-Qura University, Makkah, The Kingdom of Saudi Arabia; 2 Department of Oral Biology and Dental Anatomy, Faculty of Dentistry, Al-Azhar University, Assiut, Egypt; 3 Oral Pathology Division, Department of Basic and Clinical Oral Sciences, Faculty of Dentistry, Umm Al-Qura University, Makkah, The Kingdom of Saudi Arabia

**Keywords:** parotid gland, duct ligation, diabetes, cytokeratin 17

## Abstract

**Objectives** The present study aimed to give a glimpse of the normal distribution of intermediate filaments within the parotid gland parenchyma of mongrel dogs and to reveal the pathological changes that may occur as a result of the effects of diabetes mellitus or atrophy of the gland caused by the ligation of the excretory duct to discover whether there is a similarity in these pathological behaviors.

**Materials and Methods** Twelve healthy mongrel dogs were used in the experiment and were divided into three groups: group I (the control group), group II (dogs with alloxan-induced diabetes), and group III (dogs with the right-side duct-ligated parotid gland). The dogs were sacrificed 45 days after the parotid excretory duct were tied. The right parotid gland of all groups was dissected and prepared for histological and immunohistochemical expression of cytokeratin 17 assay.

**Results** Histological findings confirmed that the parotid gland parenchyma of the diabetic group had glandular atrophy characterized by the loss of gland structure, degenerated acini, and dilatation of the duct system. Moreover, there is a predominance of the fibrous component with the presence of fat cells within the gland compartments. On the contrary, the excretory duct-ligated group undergoes severe glandular atrophy of the previous character with the presence of duct-like structure as well as extravasation and vasodilatation. Immunohistochemical expression of cytokeratin 17 in control parotid using an immunoperoxidase technique showed that cytokeratin expression varies from negative to mild in all ducts and some serous acinar cells. The gland parenchyma of the diabetic group showed mild to strong cytokeratin expression of duct cells more concentrated in the apical part with moderate to strong expression of diffuse type in some serous acini. The intensity of cytokeratin 17 in gland compartments of the excretory duct-ligated group revealed a variation in expression that ranged from negative to strong diffuse staining throughout the gland.

**Conclusion** The severity and prevalence of cytokeratin 17 in our results are predictive of the pathological influence of both diabetes mellitus and duct ligation on the cytokeratin intracellular filaments of the salivary gland parenchyma in a different way that interferes with saliva production and/or secretion leading to xerostomia.

## Introduction

Salivary glands are exocrine glands that drain saliva into the oral cavity and play an important role in maintaining oral health. Saliva consists mainly of secretions from the submandibular (65%), parotid (23%), and sublingual (4%) glands, and the remaining 8% are supplied by the numerous minor glands. 1 Saliva performs many functions, it moisturizes the oral mucosa, facilitates speech, produces antimicrobial substances, maintains the dental integrity, and helps partly in the digestion of food. 2 3 4 The four major salivary glands in dogs include the parotid, mandibular, sublingual, and zygomatic glands, while minor glands are spread throughout the oral cavity. 5 The parotid glands of a dog are a pair of main encapsulated salivary glands of the mixed type consisting of a predominant spherical serous acini with a few scattered mucous acini. Salivary gland dysfunction is a major problem with serious adverse effects on oral health including swallowing difficulties, gum disease, and inability to eat with taste disturbances. 6 7 8 9 Also, decreased saliva secretion can lead to complications in the oral cavity by allowing excessive accumulation of bacteria leading to oral infections, overgrowth of candida that occurs particularly at the commissures of the lips, thirst (especially at night), halitosis, and rampant tooth decay. 10 11 12

Diabetes mellitus is a chronic, generalized disease characterized by high blood sugar with impaired carbohydrate metabolism. 13 Diabetes is a widespread disease associated with higher morbidity and health care costs with increased mortality. 14 According to the World Health Organization, Saudi Arabia ranks second in terms of the incidence of diabetes in the Middle East countries, with the number of diabetics approaching seven million. 15 16 17 Diabetes mellitus is a metabolic disease characterized by the deterioration of hepatocytes in type I or impaired insulin function in type II. 18 Due to the high incidence of diabetes mellitus in humans, induction of diabetes in animal models has occurred on a large scale to study this disease. A single injection of alloxan or streptozotocin to an animal leads to an elevated glucose level and low plasma insulin level causing insulin-dependent diabetes syndrome. 19 Diabetes mellitus is the most common disease that damages the salivary glands by altering their tissue structure and mechanism of saliva secretion resulting in dry mouth. 20 21 In general, diabetic animals showed various salivary gland disorders including the increase in matrix metalloproteinase-8 levels, decreased acinar volume, growth retardation, and weight loss in both the parotid and sublingual glands. 22 23 Also, it has been suggested that hyperglycemia is associated with decreased salivation and elevated salivary glucose, especially in cases of severe insulin deficiency. 24

Ligation to the parotid gland duct has contributed to the understanding of the pathology of duct obstruction which ultimately leads to progressive atrophy of the affected gland. 25 26 The causes of duct obstruction are many, which may be due to salivary stones and diseases such as chronic obstructive parotitis, parotid tumors, and exposure to radiotherapy. 27 28 29 30 On the contrary, salivary gland atrophy can occur by removing the sympathetic or parasympathetic nerve without duct obstruction. 31 Duct-ligated gland showed inflammatory cell infiltration at the onset of tissue injury (1–16 hours). 8 32 At a quantitative scale, the clogged gland undergoes rapid and progressive acute atrophy up to an absolute loss of more than 85% of lethal tissue by 2 weeks. 29 Gland atrophy was manifested by significant changes including emptying of acinar cells, dissociation, and reduction in the number and size of secretory granules. The remaining intralobular epithelium consists of atrophic acini and numerous duct-like structures with an enlarged lumen. 33 34 35

Immunohistochemistry is a technique for detecting an intracellular constituent (antigen) by antigen–antibody reaction. An antigen (immunogen) bear one or more antibody-binding sites, which are highly specific regions termed as epitopes. 36 IgG is the antibody used for immunohistochemistry produced by immunizing an animal with purified specific antigen (immunogen). The animal produces a humeral immune response to this immune factor and produces a specific antibody called a monoclonal antibody that can be isolated from the animal for use in intracellular expression of this specific antigen. 37 Cytokeratin intermediate filaments are a family of related proteins encoded by different genes and expressed in different epithelial cells. 36 Cytokeratin constitutes an important biomarker because it is stable, relatively resistant to hydrolysis, formalin-fixed, and paraffin-embedded. Also, cytokeratin intermediate filaments show great fidelity in expression and are highly antigenic. 38 Distribution of cytokeratin 17 of parotid gland correlates with intercalated, striated, and excretory duct cells, while acinar cells have weak or no cytokeratin expression in their cytoplasm. 39

Our study aimed to determine the distribution of cytokeratin within the parenchymal elements of the parotid gland of diabetic animals compared with the atrophic gland due to duct ligation to study whether the effect of diabetes mellitus leads to explicit atrophy of the gland.

## Materials and Method

### Animals and Grouping

Twelve male mongrel dogs (9–11 kg weight and 2–2.6 years old) were included in the experiment. The dogs were fed boiled horse meat, bread, milk, and water, and they were also kept under observation for 7 days before the start of the experiment to ensure that they were not infected with rabies. The dogs were divided into three groups of four animals in each, the first group being the control group, the second group being dogs with alloxan-induced diabetes, and the third group being a group of parotids with ligated ducts.

### Induction of Diabetes

Dogs of the second group were fasted for 12 hours and then injected intravenously by fresh preparation with a single dose of 100 mg/kg body weight of alloxan monohydrate (Sigma Chemical Company) dissolved in physiological saline (0.9% NaCl). Six hours after alloxan injection, the blood glucose level was measured every 4 hours until the hypoglycemic state was resolved. If blood glucose levels were too low, glucose solution (5–10%) was injected intravenously. 40 Ten days later, blood glucose concentration was determined using an enzymatic colorimetric assay based on the trend reaction. Animals that presented a glucose level at or above 200 mg/dL were included in the diabetic group in the experiment. On the contrary, the glucose level was checked at different intervals every 15 days (three times for 45 days) to ensure that diabetes persists.

### Parotid Duct Ligation

One day before the operation, dogs of the third group were given 5 g neomycin and 5 g streptomycin orally in three intermittent doses. The dogs were washed, dried, and the hairs of the cheeks and neck were shaved. Dogs were pre-anesthetized by intramuscular injection of 0.04 mg/kg atropine sulfate 10 minutes prior to anesthesia. The animals were anesthetized by the intravenous injection of 2.5% thiopental sodium at a dose of 20 to 30 mg/kg body weight. This was followed by an intramuscular injection of 0.3 mg/kg of Flexiedil to relax the laryngeal muscle and facilitate intubation. Two centimeters incision was made on the line connecting the corner of the mouth to the posterior margin of the meniscus above the masseter muscle on the right side. The flap was removed to expose the masseter muscle and the easily visible parotid duct was clamped and tied by non-resorbed silk. The fascia over the ligated duct was closed by catgut suture and the overlying skin by the interrupted suture. Post-operatively, animals were intramuscularly injected with 5 mg/kg ampicillin and 3 mg/kg gentamycin twice a day for 2 days.

### Tissue Preparation

Dogs were sacrificed 45 days after the operation, and the parotid gland was extracted and fixed in Bowen's fixative for 3 days. Fixed tissues were washed and then dried with ascending degrees of alcohol and infiltrated with molten paraffin wax to build up a block. Serial tissue sections 5 μm thick were mounted on a glass slide to be stained by hematoxylin and eosin for routine histological examination.

### Cytokeratin Immunostaining

Paraffin sections of 5 μm thickness mounted on a slide coated with poly L-lysine were immersed in 0.3% water/methanol for 30 minutes to block endogenous peroxidase activity. Then, the section was rinsed with phosphate-buffered saline and incubated with an anti-cytokeratin 17 E3 monoclonal antibody (Sigma Chemical Company) using the streptavidin-biotin labeled method with hematoxylin counterstain. The staining reaction appeared as a brownish coloration reflecting the intracellular distribution of cytokeratin 17 mesenchymal filaments within the parenchymal compartments. Staining intensity was assessed semiquantitatively and scored as negative (0), weak (1), light (2), medium (3), and strong (4).

The collected data were analyzed using the SPSS statistical package, version 23 (IBM Inc., Chicago, IL, United States). Quantitative data were calculated as the mean, standard deviation, and ranges when their distributions were found to be parametric by means of the normality test. Comparison between groups was performed using one-way analysis of variance test that was used to determine significant differences among all groups. In addition, Tukey honestly significant difference and Dunnett T3 were performed to find the significant difference in the mean values.

## Results

### Histological Findings

Clinically, there was a severe reduction in the parotid gland size of duct-ligated group in relation to both control and diabetic dogs due to which dissection became difficult from the surrounding tissue.

**Fig. 1:**
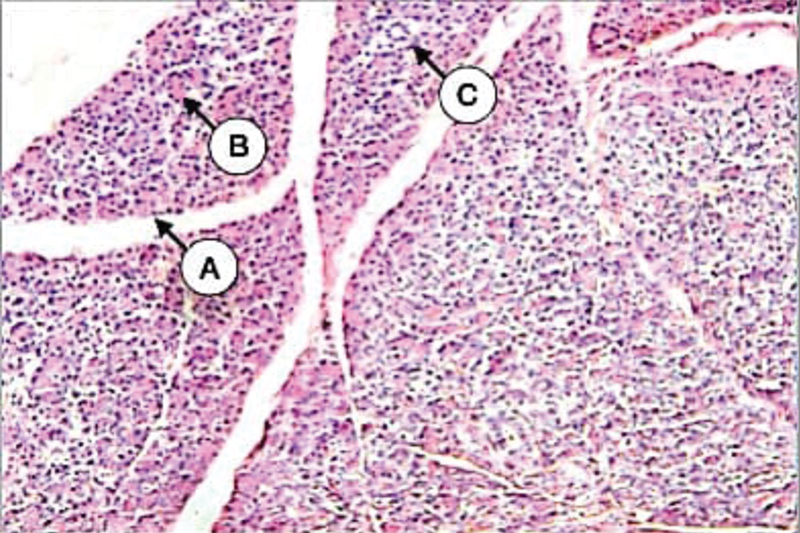
Parotid gland of control group showing definite gland lobules ( **A** ) contain spherical serous acini ( **B** ), intralobular duct ( **C** ) (H&E X 100).

The histological examination of the control parotid gland revealed the presence of numerous closely packed serous acini interspersed with numerous isolated tubular mucous acini and including intercalated, striated, and excretory ducts with individual characteristics of each. These parenchymal elements were supported by a connective tissue stroma that divides the gland into lobules and lobules ( Fig. 1 ).

**Fig. 2:**
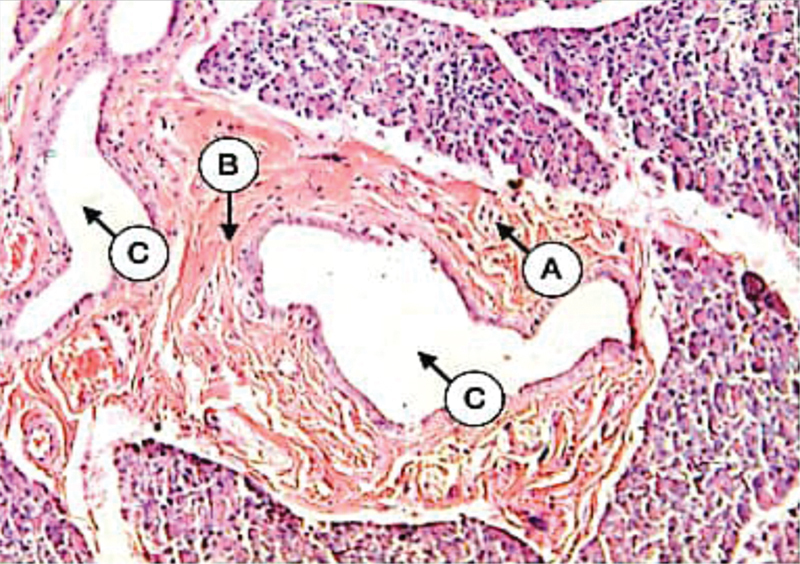
Parotid gland of diabetic dogs showing the atrophy of serous acini ( **A** ), fibrosis ( **B** ), dilated interlobular ducts ( **C** ) (H&E X 100).

The parotid glands of alloxan-induced diabetes revealed an atrophic change characterized by a decrease in parenchymal elements accompanied by an increase in the amount of fibrous tissue stroma. The acini decreased in size with the loss of normal arrangement and consisted of a group of smaller cells with an indefinite lumen. In many areas, acini were replaced by fat cells. The acinar cells decreased in size with eosinophilic cytoplasm and deeply stained enlarged small nuclei. Moreover, there was an increase in the amount of persistent mucosal acini compared with the control ( Fig. 2 ).

**Fig. 3:**
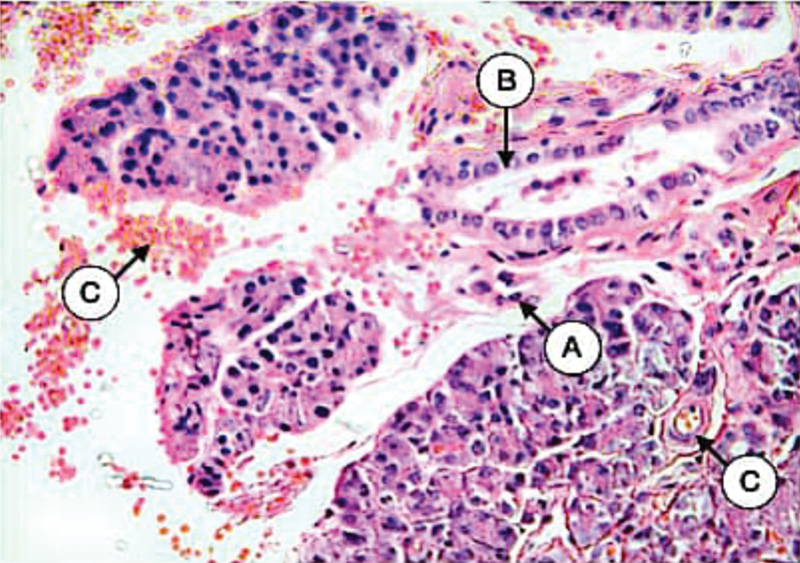
Parotid gland of duct-ligated dogs showing the atrophy of serous acini ( **A** ), dilated duct ( **B** ), duct-like structure ( **C** ), extravasated blood ( **D** ) (H&E X 200).

The duct-ligated parotid gland showed a range of pathological changes varied from a decrease in gland size to severe atrophy of the gland compartments. Most parenchymal elements were atrophied without definite acinar arrangement. The remaining acinar tissues were smaller, interspersed, and scattered through more condensed fibrous tissue capsules. The lobules appeared to contain many duct-like structures. All remaining ducts were seen dilated and surrounded by remnants of acinar cells ( Fig. 3 ).

### Immunohistochemical Findings

**Fig. 4:**
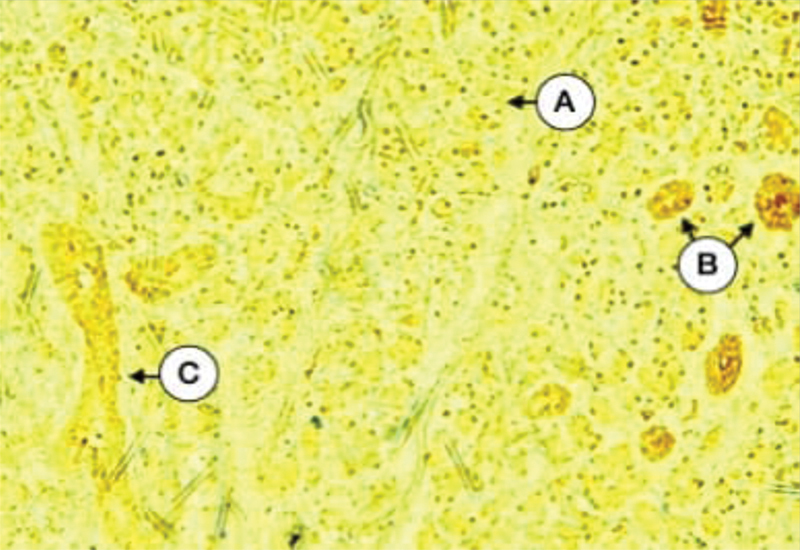
Parotid gland of control dogs showing the negative expression of CK17 in most of serous acini ( **A** ), mild in little of them ( **B** ), and weak of striated duct ( **C** ) (X 200).

The examination of tissue sections of control parotid glands incubated with the anti-cytokeratin E3 antibody against cytokeratin 17 showed varied expression of weak, mild, and moderate intensities in both intercalated and striated duct cells (data are written for each sample separately from four different tissue sections in Table 1 ). Cytokeratin 17 expression of serous acinar cells of most gland sections was negative except for some scattered acini with weak positive expression ( Fig. 4 ). The staining pattern was collected lateral and basal to the nucleus with the negative arrangement of intermediate filaments within the apical part of the cell cytoplasm allowing for free movements of secretory granules. On the contrary, several fields showed a diffuse staining pattern for cytokeratin 17 in both duct cells and serous acinar cells that indicated the resting secretory state. The main excretory ducts of several sections showed moderate expression collected within the basal cell layer with weak expression in the remaining layers. Mucous acini showed negative staining reaction in all samples.

**Table 1:** Cytokeratin intensity upon animals of all groups, negative staining (0), trace or weak (>0), mild (>1), moderate (>2), and strong staining (>3)

Intensity of CK 17 expression in duct cells															
Dogs	Control group					Diabetic group					Duct-ligated group				
	Field number					Field number					Field number				
	a	b	c	d	Average	a	b	c	d	Average	a	b	c	d	Average
1	1	2	1	1	1.25	2	1	2	1	1.5	3	1	2	2	2
2	2	1	1	1	1.25	3	2	2	1	2.00	1	2	2	4	2.25
3	0	2	2	1	1.25	3	3	2	2	2.50	2	1	3	4	2.5
4	1	1	1	0	0.75	1	2	2	2	1.75	2	3	1	2	2
	Mean ± SD				1.125	Mean ± SD				1.9375	Mean ± SD				2.1875
Intensity of CK 17 expression in acinar cells															
1	1	0	1	0	0.50	1	2	0	1	1.00	0	2	1	2	1.25
2	0	1	1	1	0.75	1	1	2	1	1.25	1	1	2	3	1.75
3	1	1	0	2	1.00	1	2	2	0	1.25	2	1	1	2	1.50
4	0	0	2	1	0.75	1	1	0	2	1.00	3	1	2	1	1.75
	Mean ± SD				0.75	Mean ± SD				1.125	Mean ± SD				1.5625

Abbreviation: SD, standard deviation.

**Fig. 5:**
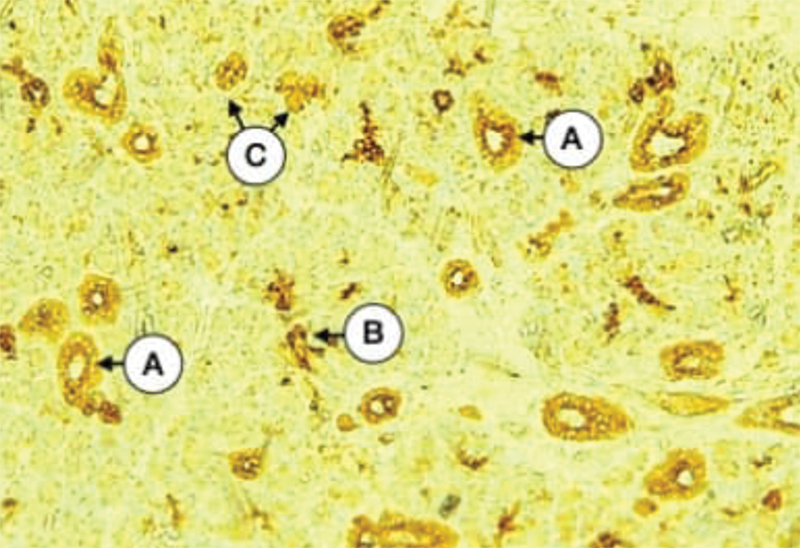
Parotid gland of diabetic dogs showing the strong expression of CK17 in ducts ( **A** ) and serous acini ( **B** ), and moderate in other serous acini ( **C** ) (X 200).

Tissue sections of the parotid glands of diabetic group incubated with the anti-cytokeratin E3 antibody against cytokeratin 17 showed varied expression of mild, moderate, and strong intensity in intercalated, striated, and excretory duct cells. On the contrary, many of the serous acini showed mild to moderate expressions of cytokeratin 17, while all sections showed negative staining for the scattered mucous acini. The staining pattern of this group varied from strong in the apical cell region and mild in the basal part to diffuse expression throughout the cell cytoplasm ( Fig. 5 ). This intracellular arrangement of intermediate filaments is thought to interfere with the formation and/or passage of secretory granules. The statistical data indicate a strong significant difference ( *p* < 0.05) for cytokeratin expression within the gland parenchyma between the diabetic group and the control group ( Tables 2 , 3 , 4 , 5 ).

**Table 2:** Multiple comparisons of CK17 in duct cells of different groups

	(I) Groups	(J) Groups	Mean difference (I-J)	Std. error	Sig.	95% Confidence interval	
						Lower bound	Upper bound
Tukey HSD	1.00	2.00	− 0.81250 a	0.22438	0.014	− 1.4390	− 0.1860
		3.00	− 1.06250 a	0.22438	0.003	− 1.6890	− 0.4360
	2.00	1.00	0.81250 a	0.22438	0.014	0.1860	1.4390
		3.00	− 0.25000	0.22438	0.530	− 0.8765	0.3765
	3.00	1.00	1.06250 a	0.22438	0.003	0.4360	1.6890
		2.00	0.25000	0.22438	0.530	− 0.3765	0.8765
Dunnett T3	1.00	2.00	− 0.81250	0.24738	0.059	− 1.6640	0.0390
		3.00	− 1.06250 a	0.17305	0.002	− 1.6153	− 0.5097
	2.00	1.00	0.81250	0.24738	0.059	− 0.0390	1.6640
		3.00	− 0.25000	0.24474	0.688	− 1.1013	0.6013
	3.00	1.00	1.06250 a	0.17305	0.002	0.5097	1.6153
		2.00	0.25000	0.24474	0.688	− 0.6013	1.1013

Abbreviation: Tukey HSD, Tukey honestly significant difference.

a The mean difference is significant at <0.05.

**Table 3:** Multiple comparisons of CK17 in acinar cells of different groups

	(I) groups	(J) groups	Mean difference (I-J)	Std. error	Sig.	95% Confidence interval	
						Lower bound	Upper bound
Tukey HSD	1.00	2.00	− 0.37625	0.14155	0.062	− 0.7715	0.0190
		3.00	− 0.81250 a	0.14155	0.001	− 1.2077	− 0.4173
	2.00	1.00	0.37625	0.14155	0.062	− 0.0190	0.7715
		3.00	− 0.43625 a	0.14155	0.032	− 0.8315	− 0.0410
	3.00	1.00	0.81250 a	0.14155	0.001	0.4173	1.2077
		2.00	0.43625 a	0.14155	0.032	0.0410	0.8315
Dunnett T3	1.00	2.00	− 0.37625	0.12542	0.071	− 0.7900	0.0375
		3.00	− 0.81250 a	0.15729	0.006	− 1.3186	− 0.3064
	2.00	1.00	0.37625	0.12542	0.071	− 0.0375	0.7900
		3.00	− 0.43625	0.14013	0.069	− 0.9142	0.0417
	3.00	1.00	0.81250 a	0.15729	0.006	0.3064	1.3186
		2.00	0.43625	0.14013	0.069	− 0.0417	0.9142

Abbreviation: Tukey HSD, Tukey honestly significant difference.

a The mean difference is significant at <0.05.

**Table 4:** Descriptive statistics of cytokeratin 17 of different groups

	* N*	Mean	Standard deviation	Standard error	95% Confidence interval for mean		Minimum	Maximum
					Lower bound	Upper bound		
1.00	4	1.1250	0.25000	0.12500	0.7272	1.5228	0.75	1.25
2.00	4	1.9375	0.42696	0.21348	1.2581	2.6169	1.50	2.50
3.00	4	2.1875	0.23936	0.11968	1.8066	2.5684	2.00	2.50
Total	12	1.7500	0.55391	0.15990	1.3981	2.1019	0.75	2.50
Descriptive statistics of CK17 expression in acinar cells of different groups								
1.00	4	0.7500	0.20412	0.10206	0.4252	1.0748	0.50	1.00
2.00	4	1.1263	0.14580	0.07290	0.8943	1.3582	1.00	1.25
3.00	4	1.5625	0.23936	0.11968	1.1816	1.9434	1.25	1.75
Total	12	1.1463	0.39119	0.11293	0.8977	1.3948	0.50	1.75

**Table 5:** One-way analysis of variance test of CK17 expression of different groups

**One-way analysis of variance test of CK17 expression in duct cells**					
	**Sum of squares**	**df**	**Mean square**	**F**	**Sig.**
Between groups	2.469	2	1.234	12.259	0.003
Within groups	0.906	9	0.101		
Total	3.375	11			
One-way analysis of variance test of CK17 expression in acinar cells					
Between groups	1.323	2	0.661	16.504	0.001
Within groups	0.361	9	0.040		
Total	1.683	11			

**Fig. 6:**
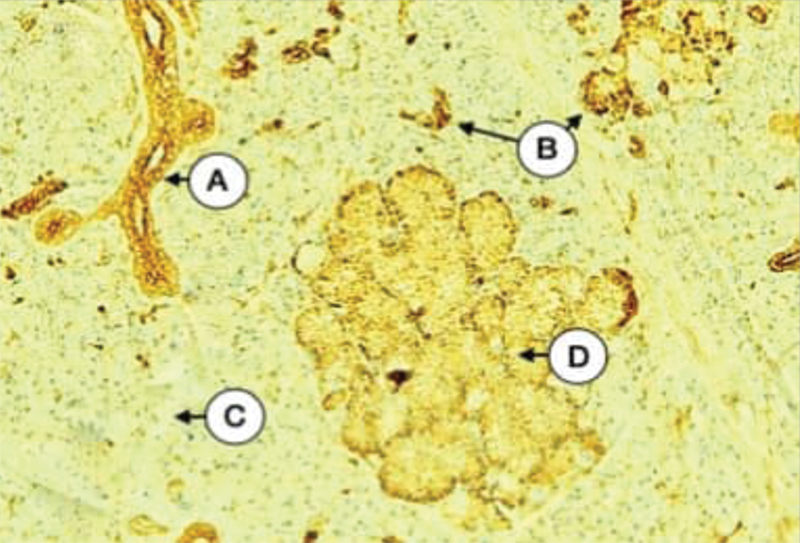
Parotid gland of excretory duct-ligated dogs showing the strong expression of CK17 in both striated duct ( **A** ), some serous acini ( **B** ), negative in other ( **C** ), and moderate in islands of mucous acini ( **D** ) (X 200).

**Fig. 7:**
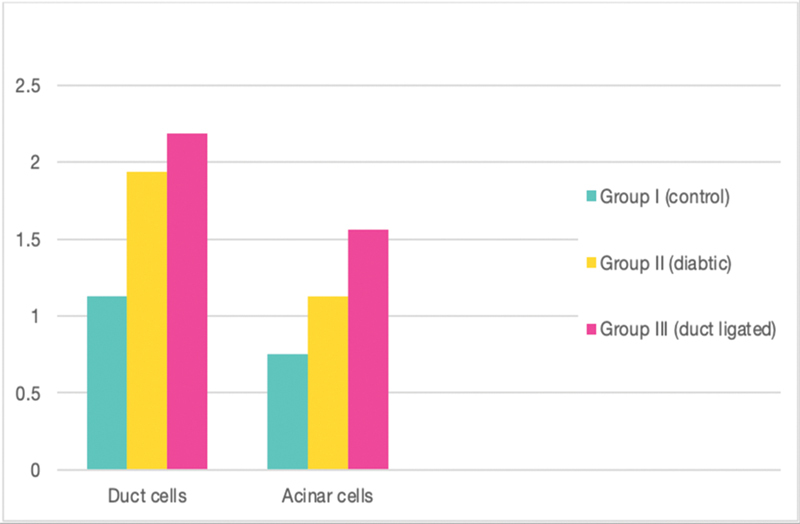
Expression of CK17 in both acinar and duct cells of all groups.

Parotid gland parenchyma of duct-ligated group incubated with the anti-cytokeratin E3 antibody against cytokeratin 17 showed widely variable expression ranging from negative to strong intensity of diffuse type. In most sections, both duct cells and serous acinar cells expression of cytokeratin 17 varied from mild, moderate, to strong intensity ( Fig. 6 ). Few sections revealed the strong expression of cytokeratin in a lot of serous acini and intercalated ducts ( Fig. 7 ). The statistical data refer to significant difference ( *p* < 0.05) for cytokeratin expression within the gland.

## Discussion

It is known that pathological changes in the parotid glands are a consequence of both diabetes mellitus and obstruction of the excretory duct. The results of the present work reported that both diabetes and duct ligation cause atrophy of the parotid gland parenchyma due to the interaction of different tissues with alterations in the processes of tissue maintenance and renewal leading to xerostomia. Pathological changes of diabetes mellitus range from a decrease in gland size to atrophy of the parenchyma of the gland which has been replaced by fibrous and/or fatty tissue with the proliferation of duct-like structures. Caldeira et al (2005) note that these histological changes were detected in all diabetic animals, both controlled and uncontrolled. 20 The persistence of multiple viable acini along with the atrophied one in the diabetic group can be considered as a sophisticated defense mechanism that maintains the acinar function of the gland parenchyma but with reduced secretory capacity. Furthermore, Mata et al (2004) reported that the persistent acini found in the diabetic gland tissues have been suggested to participate in the ability of the gland to regenerate. 21 The interpretation of our study is unable to make any attempt to distinguish the original glandular duct from the duct-like structures present in both diabetic and duct-ligated groups. This finding was supported by several authors who stated that the duct-like structures were found due to the proliferation of the duct system. 1 41 In contrast to the atrophic changes of the parenchymal elements, the connective tissue reacts through a proliferative activity, illustrating the differences in the tissue interaction of both epithelial and connective tissues. On the other hand, fibrosis present in the diabetes group and not present in the excretory duct ligation glands may indicate that the effect of diabetes may be final and irreversible, in contrast to the other, where the gland is supposed to begin the repair process once the cause of ligation is gone. Also, inflammatory changes within the connective tissue were recorded by several authors in contrast to the absence of any inflammatory cells in our study which may be related to the long time elapsed after the induction of diabetes. 4 42

It was evident in my results that the expression of cytokeratin 17 in the parotid gland of control group was clear in the duct system while it was reduced in the serous acini while the mucous acini showed negative staining. These observations may be due to highly differentiated acinar cells with a less quantities of intermediate filaments compared with duct cells. Several authors agree with this finding that the intermediate filament cytokeratin17 in salivary gland cells plays an important role in cell structure and the intensity of expression is closely related to the differentiation status of epithelial cells with further expression in duct cells. 37 The staining pattern was either diffuse or collected lateral and basal to the nucleus which is in agreement with several authors. 37 These different patterns explain the distribution of intracellular cytokeratin which is thought to be related to the functional activity of the gland where the diffuse pattern of staining indicates the resting phase, while the cytokeratin devoid in the luminal part was related to the active secretory state, these findings were supported by Friedrich et al. (2000). 43 Moreover, the pattern of cytokeratin expression focused on the basal cell end may be associated with an increase in the tensile force of acinar cells facing contractile myoepithelial cells resulting in an increased squeezing capacity to drive saliva through the lumen into the duct system.

Parotid parenchyma of both diabetic and duct-ligated groups revealed significant expression of cytokeratin 17 immunogenicity in both acinar and ductal cells with two different profiles, apically collected and diffuse. The intensity of cytokeratin expression was significantly increased toward the parenchymal elements of group III. It is thought that the luminal distribution patterns of cytokeratin in the diabetic dog may interfere with the secretory capacity of acinar cells resulting in dry mouth. Also, this luminal pattern within the ductal cells may disturb the modulation procedure for the secreted primary saliva. On the contrary, the diffuse pattern of cytokeratin expression indicates a cellular deleterious effect throughout the parenchymal compartments. The interpretation of these two different expressions may be due to the progression of the degeneration stage, which starts from the luminal side and then progresses to the diffusion pattern. However, the expression of cytokeratin 17 in the duct cells of all groups is found not only in the largest but also in the smallest ones. This observation may support the accuracy of this immunoprecipitation process for intermediate filament.

## Conclusion

The expression and arrangement patterns of cytokeratin 17 in our results predict the pathological effect of both diabetes mellitus and duct ligation on the intracellular filament system of the salivary gland parenchyma in a different way that interferes with saliva production leading to dry mouth.
